# Impacts of Microplastics on Soil Characteristics, Microbial Diversity and Greenhouse Gas Emissions: A Review

**DOI:** 10.3390/microorganisms14102221

**Published:** 2026-10-02

**Authors:** Yang Liu, Yang De, Wenhao Zhang, Chengyu Wang, Haiou Shen, Yong Yu

**Affiliations:** 1 College of Resource and Environmental Science, Jilin Agricultural University, Changchun 130118, China; liuyang17@jlau.edu.cn (Y.L.); dengyang@mails.jlau.edu.cn (Y.D.); z13844791192@163.com (W.Z.); chengyuw@jlau.edu.cn (C.W.); 2 Northeast Institute of Geography and Agroecology, Chinese Academy of Sciences, Changchun 130102, China

**Keywords:** microplastics, soil characteristics, microbial diversity, GHG emissions, carbon cycling

## Abstract

Soil microplastics (MPs) pollution has emerged as a global concern, yet its integrated impacts on soil biogeochemical cycles and greenhouse gas (GHG) fluxes remain incompletely understood. This review synthesizes studies from 2019 to 2026 on MP interactions with soil physicochemical properties, microbial communities, and GHG dynamics, emphasizing carbon and nitrogen cycling. MPs actively modify soil processes rather than acting as inert particles. Fiber and film MPs alter pore architecture, bulk density, and water retention, affecting aeration and microbial habitats. For carbon cycling, MPs modify DOC, MBC, and EOOC pools and trigger polymer-specific priming effects that range from positive to negative depending on polymer composition, with net outcomes varying across studies. For nitrogen cycling, MP-induced shifts in NO_3_^−^-N and NH_4_^+^-N availability and in nitrification and denitrification pathways yield variable N_2_O responses; notably, certain biodegradable polymers suppress N_2_O emissions in a dose-dependent manner, contrasting with the neutral or stimulatory effects of conventional plastics. Microbial communities shift functionally, with enrichment of plastic-degrading taxa, increased Proteobacteria, reduced overall bacterial abundance, and promoted fungi, while the plastisphere forms a distinct habitat from bulk soil. GHG responses are highly context-dependent, varying with polymer type, concentration, shape, aging, soil properties, and experimental conditions, and should not be generalized. Future research should prioritize environmentally relevant doses, polymer mixtures, long-term field experiments, co-pollutant scenarios, and integration of MP-derived degradation products into carbon models. Addressing these gaps will advance understanding of MP–soil–climate interactions and support evidence-based risk assessment.

## 1. Introduction

Microplastics (MPs, <5 mm) have been recognized as an emerging and widespread anthropogenic contaminant in soil ecosystems. Relevant data indicate that global plastics production reached approximately 368 million tonnes in 2019 [1], and this output is increasing annually. At this rate, an additional approximately 33 billion tonnes of plastic could accumulate on the planet by 2050, with approximately 26 to 50 million metric tonnes of plastic waste expected to be landfills or discharged into the environment [2]. MPs primarily originate from primary and secondary sources. Primary MPs are mainly used as raw materials in specific industrial processes or in the production of cosmetics [3]. Secondary MPs, on the other hand, typically result from the fragmentation of larger plastic fragments through physical, chemical or biological degradation processes [4]. MPs enter the soil environment primarily through pathways such as agricultural plastic mulching in greenhouses, application of sewage sludge, irrigation with wastewater, landfills, tire wear, and atmospheric deposition [5]. In urban and industrial soils, MPs are primarily attributed to improper disposal of waste from daily activities and industrial wastewater and waste, establishing a positive correlation between MPs concentration and population density [6]. Relative to urban and industrial soils, agricultural soils exhibit higher concentrations of microplastic particles. To address the challenges of inadequate heat accumulation and insufficient yield associated with winter crops, the utilization of greenhouses and mulch films has been extensively implemented globally [7].

The types of MPs found in agricultural soil primarily consist of non-degradable MPs such as polyethylene (PE), polystyrene (PS), polypropylene (PP), polyvinyl chloride (PVC), and polyethylene terephthalate (PET), as well as degradable MPs like polylactic acid (PLA), polycaprolactone (PCL), and polybutylene succinate (PBS) [4,8]. As MPs continuously accumulate in soil ecosystems, they have the ability to alter the physicochemical conditions of the soil, including soil water-holding capacity, aggregation, bulk density, porosity, pH value, enzyme activity, and nutrient content [9,10]. MPs have significant effects on the structure and function of soil microbial communities [11]. These changes are primarily influenced by the type, concentration, shape, and size of the MPs, as well as the inherent properties of the soil itself [10,12]. Additionally, MPs have the potential to be ingested by soil fauna, causing damage to their nervous, digestive, immune, and reproductive systems. They can also enter the human body through the food chain, ultimately posing a threat to human health [13,14]. MPs entering the terrestrial environment may affect plant germination rate, root system growth, photosynthesis, and the efficiency of nutrient utilization [15]. Alterations in terrestrial ecosystems may directly or indirectly impact the carbon–nitrogen cycle and the emission of greenhouse gas (GHG), thereby exerting an influence on global climate [16,17].

To date, existing review articles related to soil MPs have primarily concentrated on aspects such as detection methods, geographical distribution, pollution status, combined pollution and toxicological effects [4,13,18]. Synthesis specifically targeting MPs–soil–GHG linkages remains limited. The present review provides a process-oriented narrative synthesis that integrates evidence across soil physicochemical changes, microbial community and functional responses, carbon and nitrogen transformations, and GHG emissions within a common interpretative framework. To examine these linkages, this review surveys the current evidence across three dimensions: (1) MP-induced alterations in soil physicochemical properties; (2) MP-driven shifts in soil microbial community structure, functional enzyme activity, and associated gene expression; (3) an integrative perspective linking MPs’ effects on soil properties and microbial ecosystems to GHG emissions. By synthesizing findings from primary studies, this review aims to clarify the mechanisms by which MPs affect GHG dynamics and to identify knowledge gaps relevant to global warming and mitigation strategies.

## 2. Materials and Methods

### 2.1. Literature Retrieval

Relevant original research articles were identified from three databases—Web of Science, ScienceDirect, and Google Scholar—covering publications from 2019 through to September 2026. Search terms combined the core keyword “soil microplastics” with each of the following thematic terms: “soil properties,” “soil community,” “soil enzyme activity,” “soil respiration,” “soil nitrous emission,” “methane emission,” “carbon dioxide emission,” and “climate change.” These combinations were adapted to each database’s search syntax. Initial retrieval yielded 6604 records (Web of Science: 2656; Google Scholar: 3537; ScienceDirect: 411). After automated deduplication using EndNote X9 (Clarivate Analytics, Philadelphia, PA, USA), 3420 unique records remained. Titles and abstracts were reviewed for topical relevance, and full texts of potentially relevant studies were assessed based on data availability and experimental clarity. A total of 304 articles were ultimately selected for this review.

### 2.2. Data Selection and Synthesis

The 120 included studies were selected to represent a range of experimental conditions, MPs types, and soil responses that were relevant to the topics covered. Studies were prioritized according to the following considerations: (1) Microplastic exposure scenarios: Controlled experiments investigating MPs alone in soil were used as the primary evidence base. Studies involving co-pollution scenarios (e.g., pesticides, herbicides, persistent organic pollutants, antibiotics, or heavy metals) were additionally considered for qualitative discussion when experimental design allowed MP-related effects to be reasonably distinguished from those of co-contaminants. (2) Quantitative data availability: Priority was given to studies that explicitly reported the mass or particle concentration of applied MPs. (3) Controlled experimental design: Preference was given to studies that included both a microplastic-free control group and at least one MP-treated group. (4) Handling of multiple treatments within studies: When a study reported multiple MP polymer types, concentrations, particle sizes, exposure durations, or soil conditions, treatment-level information was extracted separately. All data from the same publication were treated as originating from one study and were not counted as independent replicates. (5) Synthesis approach: Findings were organized thematically across soil physical properties, chemical properties, microbial communities, and GHG emissions. No statistical pooling, meta-analysis, or effect-size calculation was performed.

### 2.3. Data Analysis

The concentration units of MPs in the literature are primarily items ·kg^−1^, g·kg^−1^, and w·w^−1^. To facilitate comparison of MPs concentrations across different studies, in the summary tables, concentrations are presented as mass-based data mainly in % (*w*/*w*) as originally reported, where 1% *w*/*w* is equivalent to 10 g MP per kg dry soil. To facilitate cross-study comparability, Equation (1) [19] provides an optional estimation method for converting the reported mass-based concentrations to particle-number concentrations.

(1) $$N\left(\mathrm{items}\;\cdot kg^{-1}\right)=\frac{C_{m}\left(g\cdot \;kg^{-1}\right)}{D\left(g\cdot \;{\mathrm{cm}}^{-3}\right)\times V\left({\mathrm{cm}}^{3}\right)}$$

where *N* is the MP particle-number concentration (items·kg^−1^); *C_m_* is the MP mass concentration (g·kg^−1^); *D* is the polymer density (g·cm^−3^); and *V* is the individual particle volume (cm^3^), calculated as *V* = *πd*^3^/6 for spherical particles, where *d* is the representative particle diameter (cm). Thus, *D* × *V* represents the estimated mass of one MP particle.

When the source data only provides values for soil organic matter (SOM), transformation is conducted using Equation (2) [20].

*SOC*(g kg^−1^) = *SOM*(g kg^−1^) × 0.58 (2)

For soil pH values in the data, when the soil pH was measured using CaCl_2_, transformation is carried out using Equation (3) [21].

*pH*[*H*_2_*O*] = 1.65 + 0.86 × *pH*[*CaCl*_2_] (3)

When the source data only provides standard error (SE), transformation is applied using Equation [22], where n is the sample size.

*SD* = *SE* × *n*^0.5^ (4)

## 3. Results and Discussion

### 3.1. Soil MPs and Soil Physical Characteristics

**Figure 1:**
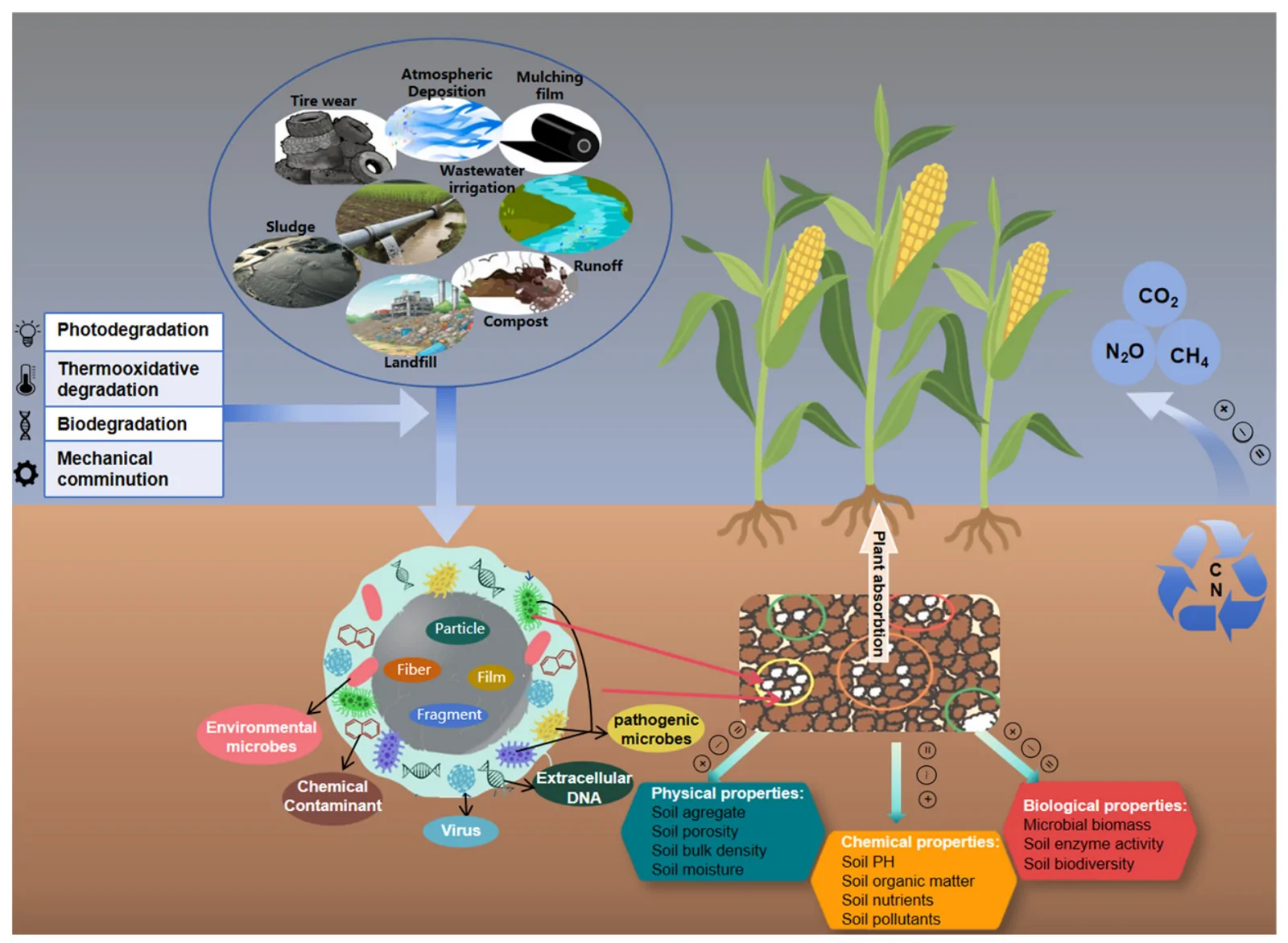
Impacts of microplastics on soil ecosystems and GHG emissions. The schematic summarizes major microplastic inputs and environmental transformation processes, along with reported or proposed changes in soil physicochemical properties, microbial communities, plant–soil interactions, and CO_2_, CH_4_, and N_2_O emissions.

Soil is a complex and dynamically changing ecosystem with numerous physical, chemical, and biological properties that influence nutrient cycling and other critical ecosystem processes [10]. MPs exhibit a complex interplay with soil characteristics, potentially binding with soil organic matter and minerals, thus becoming an integral component of the soil matrix (Figure 1) [23], with downstream consequences for soil structure, hydrology, and nutrient cycling [9,10].

Soil aggregates are central to soil structure [24,25], but MPs can diminish the aggregative capacity of soil organic matter or modify internal wet-dry cycling, impacting aggregate formation and stability [26,27]. MPs can impede the aggregation process by interacting with soil minerals and organic constituents, consequently disrupting soil structure, especially within smaller soil aggregates [28]. Furthermore, the incorporation of MPs into the soil aggregate matrix may significantly degrade aggregate stability and water-holding capacity, with an observable decline correlating with increasing MP concentrations [26,29]. MPs of different types, such as PET, PS, and polyamide (PA), have been shown to decrease water-stable aggregates and display a dose-dependent effect [30]. However, low concentrations of polyether sulfone (PES) in potting soil significantly increased water-stable macro-aggregates (>2 mm) [31]. Discrepancies among studies are common; fibers, films, foams, and fragments generally reduce soil aggregation, with fibers exhibiting the most pronounced effects [32]. This partially supports the “shape dissimilarity hypothesis,” which posits that MPs effects are more pronounced when particle shapes differ from those of soil particles [33,34]. This concept was originally proposed by Rillig and colleagues; while the broader conclusion that MP shape influences soil responses is supported by multiple independent studies (including a two-year field study showing stronger effects of shape than concentration on bacterial community assembly [35]), the specific prediction that effect magnitude increases systematically with shape dissimilarity has not yet been independently and directly tested across diverse soil types and polymer compositions. We therefore describe this hypothesis as partially supported but not established. However, the effects of MPs on soil aggregation are not solely determined by polymer properties; vegetation presence can fundamentally alter these responses. While MPs may promote macroaggregate formation in bare soils, plant roots can reverse or suppress this effect [36], likely through root-induced modifications of pore architecture, rhizosphere microbial communities, and exudate–MP interactions. Consequently, MP impacts on aggregate stability should not be interpreted solely from unplanted microcosm experiments.

Soil pore distribution plays a key role in quantifying soil structure, and the hydrophobicity of MPs can reduce water stored in small pores, thereby affecting hydraulic conductivity and solute transport [37,38]. MP fragments can disrupt the continuity of soil pores, while MP particles or powders can fill interstitial spaces within the soil matrix. These modifications alter soil bulk density, a pivotal property indicative of soil quality and fertility, and a crucial parameter for estimating soil carbon stocks at the regional scale [39]. MPs have the potential to diminish soil bulk density because their density is typically lower than that of natural minerals, and a significant correlation exists between MP concentration and bulk density [29,40]. Reduced bulk density typically increases macro-porosity and aeration [37], enhancing root infiltration, aerobic microbial proliferation, and processes such as nitrification and organic matter mineralization [24,40]. These structural changes may also influence soil pH through altered microbial N transformation. Nonetheless, certain studies have demonstrated that PES and PA MPs exert no influence on soil bulk density under specific conditions [31,40], indicating that the direction of MP effects on bulk density varies with polymer type and experimental settings. However, this variability is not limited to conventional plastics. Biodegradable polymers (e.g., PLA, PBAT) can produce divergent bulk density responses compared to conventional plastics (e.g., PE, PP, PS). Specifically, PLA and PBAT may increase bulk density or alter pore connectivity through water absorption, swelling, and binding with soil particles, contrasting with the density-driven reduction typically observed for PE [41]. This necessitates a polymer-specific rather than generalized interpretation of MPs’ effects on soil bulk density. The variability across studies, which ranges from neutral effects (PES, PA) to polymer-specific divergences (PLA, PBAT vs. PE), underscores the need for caution when extrapolating laboratory results to field conditions. Studies conducted at field-realistic MP concentrations have reported predominantly nonsignificant changes in key physical indicators, including water-stable aggregates, bulk density, and pore distribution [42,43]. This aligns with the broader caveat that nominal MP concentrations in controlled experiments typically exceed those detected in field soils; thus, while high-dose laboratory studies are valuable for probing mechanisms, their findings should not be interpreted as representative of actual field contamination scenarios.

Soil moisture determines the availability of nutrients, as well as the survival and reproduction of soil organisms and plants [44]. MP effects on water-holding capacity and evapotranspiration are polymer-specific; some (e.g., PES fragments) increase retention and evapotranspiration, while others (e.g., PE, PET, PP, PS fragments, PAA fibers, PA beads) produce nonsignificant effects [40]. Smaller MPs and higher concentrations generally amplify evaporation through channel formation [45]. Larger plastic films may induce soil cracking and exacerbate drought by promoting deeper moisture evaporation and disrupting horizontal and vertical water flow through altered aggregate size fractions and pore distribution [38,45]. MPs can also bind with soil matrices to establish moisture transport channels that accelerate evaporation, causing surface drying and cracking, which may modify soil gas diffusion and the distribution of aerobic and anaerobic microsites [24,45]. It should be noted that oxygen availability was not directly measured in most of these studies; therefore, this relationship should be regarded as a possible mechanism rather than a demonstrated response. Importantly, changes in individual aggregate metrics should not be interpreted as equivalent changes in overall soil physical quality. For instance, increasing LDPE and PBAT concentrations enhanced aggregate stability while simultaneously increasing splash erosion and reducing saturated hydraulic conductivity [46], demonstrating that improved aggregation can coexist with impaired hydraulic functioning. The effects of different types of MPs on soil properties under various experimental conditions are summarized in Table 1.

### 3.2. Soil MPs and Soil Chemical Characteristics

Soil pH is one of the principal determinants of soil chemical properties. Studies have indicated that medium to high concentrations of PLA and PE fragments can elevate pH levels [38], whereas PE has also been shown to significantly reduce pH after a 30-day exposure period [58]. Soil pH decreases with increasing concentrations of PS and polytetrafluoroethylene (PTFE), with smaller particle sizes exerting a greater effect than larger ones [71]. Soils exposed to foam and debris types of MPs exhibit a greater increase in pH than those exposed to film and fragment shapes [33]. These findings demonstrate that MP-induced pH changes are not unidirectional; depending on polymer type, particle shape, and exposure time, MPs may increase, decrease, or cause no significant change. This variability could further impact soil nutrient availability, plant growth, and crop productivity [10,12]. The underlying mechanisms involve polymer-dependent additive leaching (e.g., alkaline fillers), MP surface adsorption of H^+^ and organic acids, MP-induced modifications to soil physical structure that alter N-cycling and the nitrification–ammonification balance, and DOM release during biodegradable polymer degradation [8,10]. The net pH effect thus reflects a balance among these sometimes opposing processes, varying with MP type, size, shape, concentration, soil properties, and exposure conditions.

Soil organic carbon (SOC) comprises carbon-containing materials derived from plant, animal, and microbial residues and metabolites [72,73]. As a key determinant of soil fertility, SOC influences soil structure, water retention, nutrient cycling, and biodiversity [72], and its fluctuations can substantially affect GHG emissions and the global terrestrial carbon balance [72]. MPs represent an additional carbon input to soils, but their contribution to soil C cycling strongly depends on polymer degradability. Conventional polymers such as PE are relatively recalcitrant and generally exhibit slow turnover, whereas biodegradable polymers such as PLA-, PBS-, and PHA-based materials can undergo microbial degradation and release bioavailable C substrates, thereby altering native SOC mineralization and priming processes [74,75]. Importantly, MP effects on SOC operate through multiple pathways. First, MPs concurrently modify soil physical properties by reducing bulk density and enhancing macro-porosity and aeration, which can, but do not invariably, accelerate native SOC mineralization via stimulated aerobic processes [24,40]. Second, MPs may reduce SOC bioavailability to microorganisms, partially offsetting mineralization losses [76]. Third, beyond these physical and bioavailability effects, the priming effect represents a distinct biochemical pathway. The priming effect refers to the acceleration or suppression of native SOC decomposition induced by exogenous substrate addition. Evidence indicates that priming responses are highly polymer-specific: PHA particles induce strong positive priming, stimulating native SOC mineralization by 200–250% [77], whereas PLA exhibits negative priming, suppressing SOC decomposition by 5% to 22% [77]. Recent field studies further demonstrate that total SOC alone may underestimate MP-induced C dynamics; PP and PLA caused substantial redistribution between plant-derived and microbial-derived C pools even when total SOC remained stable [65]. Therefore, MP effects on soil C should be evaluated across multiple fractions, including aggregate-associated SOC, easily oxidizable C, and microbial vs. plant-derived C, rather than total SOC alone [65,66,78].

Soil enzymes are closely associated with energy flow and nutrient cycling [79]. Urease, fluorescein diacetate hydrolase (FDAse), phosphatase, sucrase, and catalase serve as key indicators of soil quality [80,81], with urease reflecting soil fertility, FDAse indicating microbial metabolic activity, and catalase correlating with aerobic microorganisms and SOM dynamics. MPs alter these enzyme activities; for example, PE and PVC inhibit FDAse [81], while phosphatase activity shows a significant positive correlation with MP concentration [81]. In addition to these indicator enzymes, extracellular carbon-degrading enzymes specifically catalyze organic carbon mineralization into CO_2_, CH_4_, and H_2_O, directly linking enzyme-mediated processes to GHG emissions [47,57]. Notably, the enzymatic capacity of soil microorganisms extends to MP biodegradation. Bacteria, actinomycetes (e.g., *Streptomyces* spp.), and fungi (e.g., *Aspergillus*, *Penicillium*) colonize MP surfaces, form biofilms, and secrete hydrolases (lipases, carboxylesterases, cutinases, proteases) that cleave ester and amide bonds in biodegradable polymers (PLA, PBAT, PBS), as well as oxidases (laccases, peroxidases, phenol oxidases) that generate reactive oxygen species (ROS) to attack C–C bonds in recalcitrant polymers (PE, PS, PP) [18,82]. These extracellular enzymes depolymerize MP chains into bioavailable oligomers and monomers that are assimilated, metabolized, and ultimately converted to CO_2_ under aerobic conditions or CH_4_ under anaerobic conditions [18,82]. The efficiency of this process depends on polymer crystallinity, molecular weight, additives, microbial community composition, and soil conditions [18]. However, the specific mechanisms by which MPs affect soil enzyme activity remain incompletely understood. Further research is needed to distinguish between direct enzyme inhibition or stimulation by MP-derived compounds and indirect effects mediated through changes in soil physicochemical properties or microbial community composition.

### 3.3. Influence of MPs on Soil Microbial Community and Functional Genes

Microorganisms are the primary drivers of organic matter decomposition, pollutant degradation, and nutrient cycling in soil ecosystems [83]. MPs interact with these communities through multiple pathways. They serve as carriers that facilitate microbial vertical migration via biofilm-mediated transport, alter soil physicochemical properties and SOC content, and provide distinct ecological niches determined by polymer backbone structure, surface area, hydrophobicity, and shape [84,85]. These interactions exert selective pressure on soil microbial communities, and the outcome varies with polymer type, shape, concentration, and environmental conditions [12,35]. At the community level, MP shape can be more influential than concentration. A two-year field study found that MP shape influenced bacterial α-diversity and community assembly more strongly than concentration, with fiber and fragment shapes inducing greater changes than ball and powder shapes [35]. Bacterial β-diversity in the same experiment was also more strongly differentiated by shape than by concentration, suggesting that microbial responses do not invariably follow a simple dose-dependent pattern. Together with studies showing that MPs of similar shapes but different polymer compositions can elicit contrasting responses, these findings indicate that particle geometry, polymer chemistry, and associated material properties may be more important than nominal dose alone in some systems [32,35]. At the taxon level, fibrous and film MPs increase Proteobacteria abundance while reducing Acidobacteria [57,86,87]. Importantly, microbial responses in bulk soil should be distinguished from those occurring directly on MP surfaces. Within the plastisphere, MPs act as artificial substrates that adsorb nutrients and can be colonized by fungal and actinomycete hyphae, which bypass the surrounding soil to access plastic surfaces directly [85]; PE biofilm colonization yields lower α-diversity than surrounding soil yet enriches taxa with putative plastic-degrading capacity [88]. Moreover, fungal communities may show greater sensitivity to MP exposure than bacterial communities under certain soil conditions, such as saline-alkali soil [89]. The presence of plant roots can further modify plastisphere community assembly, as root exudates and rhizosphere microbial hotspots alter the selective environment on MP surfaces [69,85]. This plant–soil–plastic interplay implies that unplanted experimental systems may underestimate the complexity of MP-driven microbial selection in situ. Recent field evidence further suggests that the plastisphere may represent more than a localized compositional shift within the bulk-soil microbiome [90]. Plastisphere microbial networks have been reported to be simpler, more modular, and less robust than the surrounding soil networks, with differences also observed in community assembly processes and N-cycling functional genes [90]. In some environments, MP-associated pollutants can partially override the edaphic and spatial factors that ordinarily structure bulk-soil communities, producing a microbial habitat that is partly decoupled from the surrounding soil matrix [90]. Thus, MPs may create distinct ecological filters and interaction networks rather than merely altering taxon relative abundance [90]. However, neither increased nor decreased microbial diversity should be interpreted inherently as beneficial or detrimental to soil functioning [11,85]. Diversity metrics describe community structure, whereas ecosystem consequences depend on which taxa, interactions, and functional traits are gained or lost. Collectively, MP-induced microbial responses are highly context-dependent and should not be generalized as uniformly increasing or decreasing microbial abundance or diversity.

The plastisphere can selectively enrich microbial taxa putatively associated with plastic degradation, which has implications for understanding the role of MP-associated communities in plastic transformation [85,88]. Initial depolymerization of susceptible polymers can occur extracellularly through microbially secreted enzymes, whereas resulting low-molecular-weight products may subsequently be assimilated and metabolized intracellularly [18,82]. Thus, extracellular polymer cleavage and intracellular metabolism represent distinct but sequential stages of microbial MP biodegradation. These processes can, in turn, reshape soil microbial community structure, with studies reporting shifts in the relative abundance of multiple bacterial and fungal taxa in MP-amended soils [86,91]. Such compositional changes may reflect microbial adaptation to MP-associated conditions; however, taxonomic enrichment alone does not confirm enhanced degradation capacity. Demonstration of biodegradation therefore requires complementary evidence, such as polymer mass loss, molecular-weight reduction, surface-chemical changes, degradation products, isotope tracing, or direct enzymatic activity [85]. This distinction is particularly important for taxa frequently described as “plastic-degrading” on the basis of their enrichment on MP surfaces. Their relative abundance may increase because the plastisphere provides favorable habitat, nutrients, additives, or associated organic substrates rather than because the organisms actively mineralize the polymer itself. Aged MPs develop surface functional groups that facilitate microbial aggregation and may alter surface redox properties, enabling interactions with the microbial metabolism in which MPs serve as electron donors or sinks [92,93]. Because soil microorganisms both mineralize SOC to inorganic carbon and fix inorganic carbon into SOC [49,94], electron-transfer interactions with MPs that differ in electron-donating capacity from native organic matter could alter the balance between C mineralization and microbial C stabilization [95]. Recent field studies support this: in a dryland agroecosystem, inert PE intensified microbial C limitation and increased carbohydrate-degrading functional groups, coinciding with reduced particulate organic C, whereas biodegradable PLA was associated with greater C- and N-metabolic potential, enhanced amino-sugar and EPS-related pathways, and increased microbial necromass [78]. These observations indicate that similar evidence of “microbial change” can translate into fundamentally different consequences for SOC persistence depending on polymer degradability [65,78]. Biodegradable and conventional polymers differ in their electron-donating capacities and surface functionalization during aging, which may explain divergent effects on microbial electron-transfer networks and downstream carbon transformation [93,95]. Nevertheless, the specific pathways through which MPs influence soil carbon transformation remain to be fully elucidated. The ecological significance of microbial responses should ultimately be evaluated in terms of their consequences for C stabilization and transformation, rather than from diversity or gene abundance alone.

In addition to reshaping community structure and carbon metabolism, MPs can alter the abundance and expression of functional genes that regulate soil C and N cycling. Because these genes encode proteins driving key biogeochemical reactions, their abundance serves as an indicator of microbial metabolic potential rather than direct process rates [96,97]. This distinction is essential when interpreting molecular evidence: gene abundance indicates the potential size of a functional guild, whereas transcription, enzyme activity, substrate availability, and environmental constraints jointly determine whether that potential is translated into an actual biogeochemical flux. Soil C-cycle functional genes include carbon fixation genes (e.g., *accA*, *aclB*, *acsA-F*, *acsB*, *acsE*) and carbon degradation genes (e.g., *amyA*, *apu*, *sga*, *abfA*, *xylA*, *manB*, *cex*, *naglu*, *lig*, *mnp*) [49,96,98]. Carbon degradation genes encode enzymes that break down complex organic polymers such as starch (*sga*, *amyA*), hemicellulose (*abfA*, *manB*, and *xylA*), cellulose (*cex*), and lignin (*lig*, *mnp*) into readily assimilable substrates [49,96]. Through these genes, soil microorganisms decompose particulate and dissolved organic matter, releasing labile carbon that can be respired as CO_2_ or channeled into other metabolic pathways. Responses of these genes to MPs are not uniform. For instance, PE-MP addition increased *abfA* and *mnp* abundances in paddy soils in one study [96], whereas LDPE-MPs reduced several C-degradation gene abundances in another [49]. Such contrasting outcomes indicate that MPs can differentially alter the genetic potential for degrading specific organic C fractions depending on polymer type, aging status, and soil conditions. At field-realistic MP concentrations, however, changes in functional gene abundance are often non-significant, suggesting that high-dose laboratory findings may overstate genetic responses under ambient conditions [53]. Accordingly, changes in C-degradation genes abundance should not be interpreted as direct evidence of altered SOC turnover unless accompanied by measurements of enzyme activity, substrate turnover, respiration, microbial necromass formation, or source-resolved C fluxes. Beyond carbon cycling, MPs also affect functional genes involved in CO_2_ fixation, nitrogen cycling, and methane metabolism. CO_2_ fixation primarily occurs through the Calvin cycle, catalyzed by RubisCO; *cbbLR* and *cbbLG* are its functional genes, with *cbbLR* playing a more dominant role in soil microbial carbon fixation [94]. MPs may indirectly affect soil carbon fixation by altering the abundance of these genes in response to modified soil physicochemical conditions [99]. Diazotrophs maintain soil NH_4_^+^-N balance via biological N fixation and the *nifH* gene is widely used as a molecular marker of diazotrophic potential [86]. Studies have shown that *nifH* gene abundance is positively correlated with NH_4_^+^-N [100]; however, such correlations do not establish direct causation. The *amoA* gene in ammonia-oxidizing bacteria and archaea encodes the catalytic subunit of ammonia monooxygenase and serves as a marker for nitrification potential [86,101]. Studies have reported altered AOA*-amoA* and AOB*-amoA* expression in response to MP addition [102]; but these changes indicate altered potential rather than demonstrated nitrification rates, which require direct measurement of NH_4_^+^ consumption or NO_3_^−^ production. The *nirK* and *nirS* genes encode nitrite reductase and catalyze NO_2_^-^ to NO conversion, serving as markers for denitrification [96]. Changes in their abundance indicate altered denitrification potential rather than direct rates. The same interpretative limitation applies when such gene responses are used to explain N_2_O emissions: changes in *nirK* or *nirS* alone cannot determine the direction or magnitude of net N_2_O flux because N_2_O production and reduction involve multiple microbial pathways and environmental controls. Similarly, *mcrA* and *pmoA* reflect the genetic potential of methanogenic and methane-oxidizing communities, respectively, rather than direct CH_4_ production or oxidation measurements. Nevertheless, when considered together with measured CH_4_ fluxes, these genes can provide useful mechanistic evidence [52,102,103]. The direction of MP effects on N-cycling genes varies with polymer degradability; biodegradable polymers such as PLA can suppress nitrification and denitrification potential through dose-dependent inhibition, whereas conventional plastics may produce neutral or stimulatory effects [56,97,103]. This polymer-specific divergence underscores the inadequacy of generalized statements regarding MP impacts on N-transformation genes.

### 3.4. Soil MPs and GHG Emissions

GHG, including CO_2_, N_2_O and CH_4_, absorb longwave radiation emitted by the Earth’s surface and atmosphere, thereby driving atmospheric warming [16,104]. Among these, N_2_O exhibits a greater warming potential than CH_4_, which in turn surpasses CO_2_, while CO_2_ has the longest atmospheric residence time, followed by N_2_O, with CH_4_ being the shortest duration [105]. Soil emissions of these gases are closely linked to C and N transformations and can therefore be altered by MP-induced changes in soil physicochemical conditions and microbial processes [16,17]. Importantly, current evidence indicates that the effects of MPs on GHG emissions are not uniformly stimulatory but vary among gas types, polymers, exposure levels, and soil conditions (Table 2).

#### 3.4.1. Impacts of Soil MPs on Nitrous Emission and N Cycling

**Figure 2:**
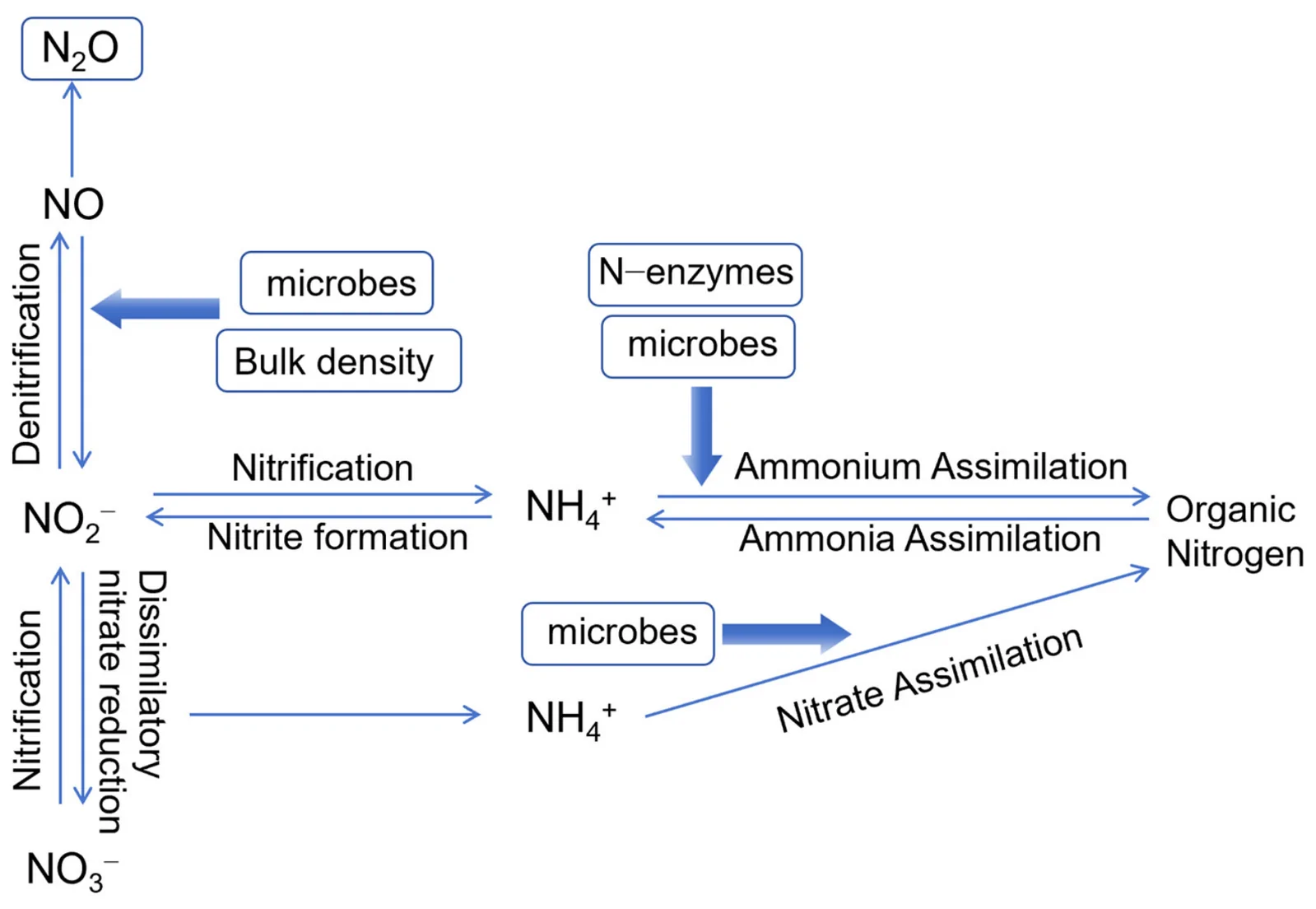
Nitrogen cycling processes in terrestrial ecosystems. The schematic illustrates transformations among organic N, NH_4_^+^, NO_2_^−^, NO_3_^−^, NO, N_2_O, and N_2_ via microbially mediated N-transformation processes, including assimilation, mineralization, nitrification, and denitrification.

Soil nitrogen is an essential nutrient for plants and drives nitrogen mineralization–immobilization. Soil N availability is regulated by mineralization and immobilization processes that control the transformation between organic and inorganic N pools. The major nitrogen cycling processes in terrestrial ecosystems, including mineralization and immobilization, are summarized in Figure 2. Mineralization converts organic nitrogen into inorganic forms (e.g., ammonium or nitrate) [111]; immobilization denotes the process whereby inorganic nitrogen is assimilated by microorganisms and reincorporated into organic nitrogen compounds, collectively regulating soil N availability and ecosystem dynamic equilibrium [111]. Direct measurements of N_2_O emissions reveal that MP effects are not consistently stimulatory. As summarized in Table 2, responses range from increases and decreases to nonsignificant effects, with both concentration- and time-dependent changes observed. For example, low PE concentrations (0.01% and 0.1%) exerted no significant effect on N_2_O emissions, whereas higher concentrations (0.5% and 1.0%) enhanced emissions [56]. In another study, PE initially suppressed N_2_O emissions but subsequently promoted them with prolonged exposure [48]. Together with more recent reports of polymer-specific decreases in N_2_O emissions, these findings indicate that the direction of the N_2_O response cannot be generalized across MP–soil systems [97,103,109].

Because N_2_O is produced through nitrification and denitrification of inorganic N substrates, MP-induced changes in NO_3_^−^-N and NH_4_^+^-N availability are central to understanding this response variability [22,111,112]. Although several studies have explored the impact of MPs on soil inorganic nitrogen availability, their findings are not entirely consistent [112]. Some studies report reductions in both NO_3_^−^-N and NH_4_^+^-N under MP exposure [12,64,101,113], whereas others report increases [114] or opposing directional shifts (e.g., NO_3_^−^-N ↓ with NH_4_^+^-N ↑ [36,115], or vice versa [116,117]). These contrasting outcomes indicate that MPs exert context-dependent effects on soil nitrogen cycling, driven by MP type, concentration, size, soil characteristics, exposure duration [112], temperature [98,115], and plants’ presence [36,97]. Such variation in NH_4_^+^-N and NO_3_^−^-N availability is directly relevant to N_2_O production because these compounds serve as substrates for nitrification and denitrification. Consequently, MP-induced changes in substrate availability, together with changes in soil moisture, aeration, pH, and redox conditions, may shift the relative contributions of N_2_O-producing and N_2_O-consuming pathways. Beyond substrate-mediated effects, the microbial “electron-shuttling” hypothesis has been proposed as a possible explanation for some MP-induced changes in soil C and N transformation. According to this hypothesis, redox-active functional groups on MP surfaces may participate in electron-transfer processes and thereby influence microbial metabolism [24]. However, direct evidence demonstrating electron transfer between MPs and soil microorganisms remains limited. Therefore, observed changes in microbial activity or nutrient availability should not be interpreted as evidence of electron shuttling; this mechanism remains a hypothesis requiring direct validation. Regardless of the underlying mechanism, empirical evidence demonstrates that MP effects on soil N_2_O emissions and N- transformation are polymer- and dose-dependent. Biodegradable polymers such as PLA can suppress N_2_O emissions in a concentration-dependent manner; for example, PLA application at 0.05% and 1% reduced N_2_O fluxes by 19.1% and 39.7%, respectively [97], likely through inhibition of nitrification and denitrification pathways [97]. In contrast, conventional plastics (e.g., PE, PP) often produce neutral or stimulatory effects on N_2_O emissions [48,56], indicating that the direction of MP impact on nitrogen transformation is not uniform across polymer types. These findings reinforce the conclusion that MP–N cycle interactions cannot be generalized and must be evaluated on a polymer-specific basis, particularly when comparing biodegradable and conventional plastics. Overall, the expanding evidence challenges the simplified view that MPs uniformly enhance soil N_2_O emissions. Low-dose null effects, polymer-specific inhibition, and temporal reversals indicate that MP-induced N_2_O responses arise from shifts among competing N-transformation pathways rather than uniform N-cycle acceleration.

#### 3.4.2. Influence of MPs on Carbon Dioxide, Methane Emission and C Cycling

**Figure 3:**
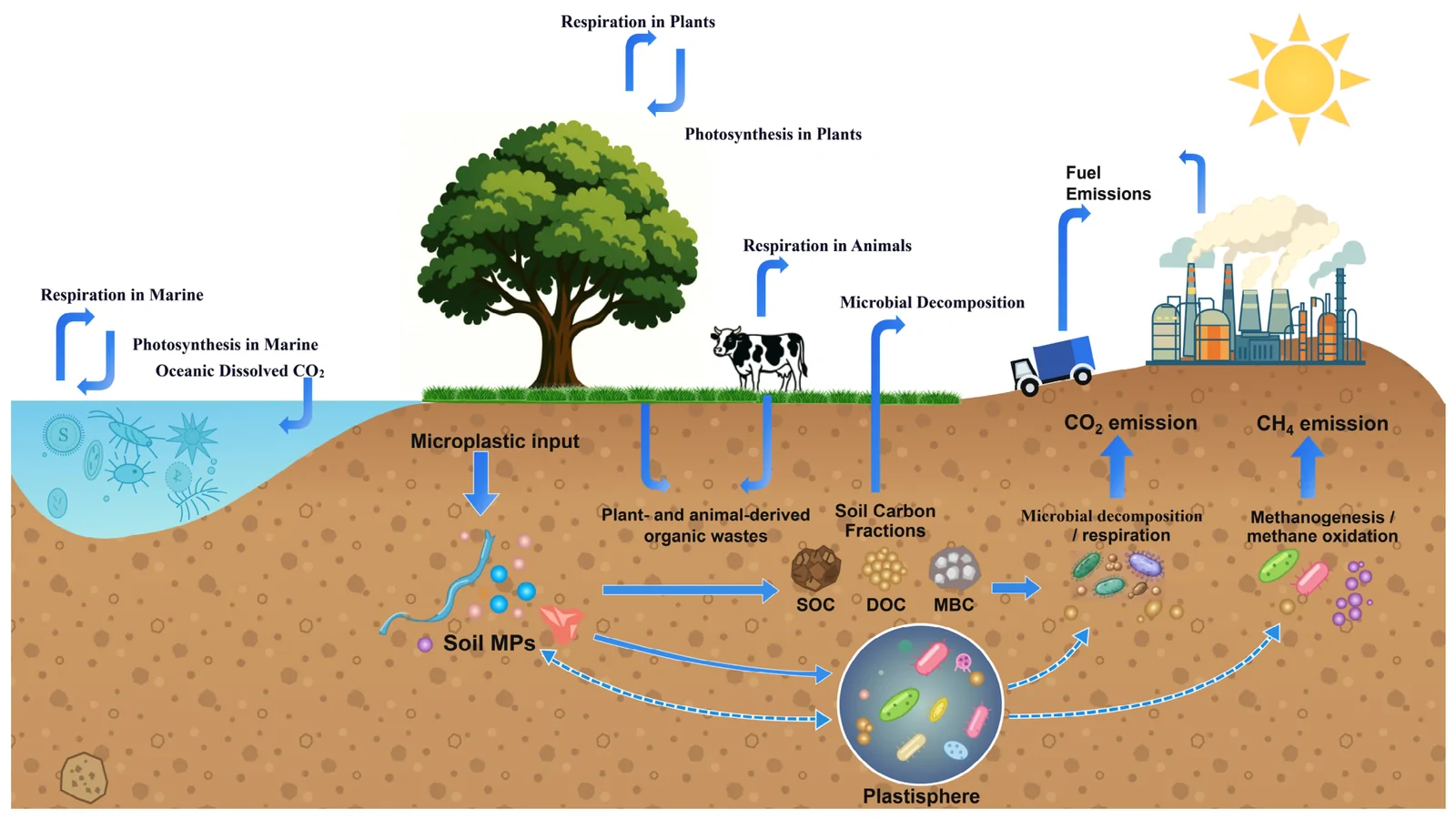
Carbon cycling processes in terrestrial ecosystems. MPs may alter soil physicochemical conditions, soil organic carbon dynamics, microbial activity, and carbon-transforming processes, thereby potentially affecting CO_2_ and CH_4_ emissions. These depicted pathways summarize reported associations and hypothesized mechanisms, and should not be interpreted as universally demonstrated causal relationships.

Major carbon cycling processes in terrestrial ecosystems, including photosynthesis, respiration, microbial decomposition, and SOC sequestration, are illustrated in Figure 3. This schematic incorporates the emerging role of soil MPs as both an exogenous carbon source and a physical stressor. By interacting with soil carbon fractions and the plastisphere, MPs alter carbon fluxes and modify CO_2_ and CH_4_ emissions [16,24,110]. The influence of MPs on CO_2_ emission flux is intricately linked to MP type, concentration, aging degree, and prevailing soil conditions [118,119]. Although dose-dependent responses have been observed, with high MP doses (1.00%) increasing soil CO_2_ production while low concentrations (0.01% and 0.10%) had negligible effects [49], this pattern should not be generalized across all polymers or soils, as stimulatory, inhibitory, and non-significant responses have all been reported. Under high-dose conditions, stimulatory effects are mediated by enhanced microbial metabolism, increased DOC content, and improved oxygen availability [24,61,120]. However, biodegradable polymers such as PLA may deviate from this pattern, inhibiting rather than stimulating CO_2_ emissions under specific conditions, possibly through negative priming effects that reduce native SOC mineralization [76,77]. Nevertheless, recent evidence summarized in Table 2 indicates that biodegradable MPs (particularly PLA, PBAT, and related blends) more frequently exhibit stronger CO_2_ release stimulation or positive SOC priming than conventional MPs, as their degradation provides additional bioavailable carbon substrates [74,108]. This apparent contradiction may reflect differences in experimental conditions (e.g., soil type, moisture, exposure duration), indicating that the net effect is context-dependent. Notably, biodegradability should not be interpreted as a climatic advantage, since more rapid degradation may simultaneously enhance C mineralization and CO_2_ release. During aging, MPs undergo substantial alterations in morphology and surface functional groups [92,121]. Aged MPs generally increase CO_2_ emissions relative to pristine counterparts by modulating microbial metabolic behavior, primarily through increased oxygen-containing functional groups and expanded adsorption area [92,95]. However, contrasting responses have also been reported [95,122], indicating that MP effects on CO_2_ emissions are context-dependent. An additional complication is the origin of the emitted CO_2_. An increase in total CO_2_ release does not necessarily indicate accelerated loss of native SOC, because the emitted C may originate from MP-derived C, native soil C stimulated through positive priming, or both. Recent source-partitioning studies have begun to distinguish plastic-C mineralization from SOC-derived CO_2_ and have demonstrated that different polymers can induce positive, negative, or negligible priming responses [74,75,77,108]. Therefore, studies measuring only total CO_2_ emissions may be insufficient to determine whether MPs accelerate native SOC loss, highlighting the importance of isotope tracing and other source-partitioning approaches.

Beyond CO_2_, CH_4_ emissions and overall global warming potential (GWP) are highly sensitive to MP properties. Existing studies report that MPs can increase, decrease, or cause no significant change in CH_4_ and CO_2_ emissions, with response direction and magnitude varying across polymer type, concentration, particle size and shape, aging status, soil properties, moisture, and redox conditions [16,52,102,103]. CH_4_ showed particularly pronounced bidirectional responses. Because net CH_4_ emission reflects the balance between microbial methanogenesis and methane oxidation, MP-induced changes in labile C availability, soil moisture, aeration, and redox conditions may shift this balance in opposite directions. Increased degradable C under anaerobic conditions may favor methanogenesis, whereas enhanced aeration or stimulated methane oxidation may suppress net CH_4_ release [52,103,123]. Meta-analyses have reported overall increases in some GHG emissions, including CH_4_ and CO_2_ [17]; however, these pooled estimates represent average responses across heterogeneous experiments and should not be interpreted as universal MP effects. Grouping chemically and biologically distinct polymers into a single “MP” category may mask polymer-specific responses, including opposite effects by different polymers on the same GHG. Furthermore, priming effects induced by biodegradable MPs complicate CO_2_ emission interpretations: positive priming (e.g., PHA) may amplify CO_2_ fluxes beyond MP carbon input alone [77], whereas negative priming (e.g., PLA) may offset or reverse stimulation [76,77]. Experimental dose and duration also require caution. Many laboratory studies employ high nominal MP concentrations that should not be assumed to represent field contamination levels (Table 2). Strong responses under high-dose conditions may therefore overestimate effects at environmentally realistic exposures. Similarly, short-term incubations may capture transient disturbances rather than long-term trajectories. Temporal reversals, such as early inhibition followed by later stimulation, indicate that MP effects evolve as particles age, microbial communities adapt, and degradation products accumulate [43,48]. These pooled estimates do not preclude opposite or null effects under specific experimental conditions [17,43,103]. In summary, current evidence does not support the assumption that MPs uniformly enhance soil C-related GHG emissions. Rather, the net response emerges from the interplay between resource stimulation (MP-derived C inputs, increased DOC availability, enhanced microbial metabolism) and microenvironmental constraints (altered soil structure, moisture, aeration, redox conditions). Which process dominates depends on polymer chemistry and degradability, exposure level and duration, and the surrounding soil environment [124]. The key question is therefore shifting from whether MPs increase soil GHG emissions to under which conditions MP effects shift between stimulation, inhibition, and negligible responses.

The SOC pool profoundly impacts the global carbon balance [72]. Among SOC fractions, dissolved organic carbon (DOC), microbial biomass carbon (MBC), and easily oxidizable organic carbon (EOOC) are the most active forms participating in biogeochemical transformations and GHG emissions [73,125]. DOC consists of glucose, organic acids, microbial biomass, and metabolites, and is highly mobile. MPs can alter soil DOC content through two primary pathways. First, MPs enhance DOC generation by increasing microbial activity and subsequent SOC mineralization [120,126]. Second, MPs promote the accumulation of high molecular weight humic substances, thereby elevating DOC levels [27]. Empirical studies support these mechanisms. For instance, PE-MPs at concentrations ranging from 0.1% to 18% alter soil microbial biomass and activity, leading to increased DOC content and affecting soil CO_2_ emissions [120]. Additionally, MPs can leach low-molecular-weight compounds into the soil solution, further modifying the DOC pool [8,95]. MBC refers to the total carbon content of all living soil microorganisms with a volume less than 5 × 10^3^ μm^3^ and reflects microbial quantity [127]. MPs influence MBC and associated carbon cycling through both direct and indirect pathways. Directly, MPs alter SOC content, which in turn modifies the carbon substrate available for microbial biomass [61,74]. Indirectly, MPs reshape soil microbial processes, including community structure, enzyme activity, and functional gene abundance [16,78]. Specifically, MPs absorb nutrients and organic matter from the surrounding environment, thereby providing ecological niches that support microbial colonization [85,88]. Conversely, MPs can release toxic additives or co-adsorbed pollutants (organic contaminants, heavy metals, antibiotics), which suppress microbial growth and alter SOC mineralization [80,128,129]. These microbial changes directly feed back to carbon transformation, as MBC participates in SOM decomposition, pollutant degradation, and nutrient cycling [125,130]. EOOC is the most active SOC component, defined as substances that can be oxidized by KMnO_4_ [66,73], and directly participates in soil biochemical transformations [66,131]. MP effects on EOOC are less explored but likely involve altered microbial oxidative capacity and labile carbon substrate availability. Current research on MP impacts on soil EOOC remains limited, highlighting a gap for future investigation. Although most MPs are relatively stable and currently constitute a minor SOC fraction, prolonged degradation may generate low-molecular-weight decomposition products that accumulate in soil carbon pools [18,54,132]. These products may interact with native SOC via priming effects, further complicating the net carbon balance [74,77]. These products could gradually integrate into labile SOC fractions, warranting the inclusion of MPs in future SOC assessments. However, MP-derived C should be distinguished from native SOC in sequestration evaluations, because incorporation of plastic-derived C into measured SOC pools does not necessarily represent additional biologically stabilized soil C [54,124,132].

## 4. Limitations and Challenges

This review has several limitations that should be considered when interpreting the results. Methodologically, this work was designed as a narrative review informed by a structured literature search; no formal risk-of-bias assessment or quantitative effect-size synthesis was performed, and evidence was not weighted by study quality. A further constraint lies in the evidence base itself: most data were derived from controlled laboratory or pot experiments, where nominal MP concentrations often exceeded field-detected levels by 1–3 orders of magnitude, limiting direct extrapolation to field soils. Additional uncertainty arises in linking MP-induced changes in soil structure, microbial activity, and functional potential to actual GHG emissions, as gene-level microbial shifts do not always correspond to measurable biogeochemical process rates. Moreover, this review focused exclusively on single-factor MP exposure, and studies involving co-pollution scenarios were excluded. However, in real agricultural soils, MPs commonly coexist with other contaminants (heavy metals, antibiotics, pesticides), and such interactions may alter both the magnitude and direction of MP effects on soil properties, microbial communities, and GHG emissions. Finally, the evidence base relies predominantly on short-term laboratory studies, limiting inference regarding long-term MP aging processes and field-realistic response patterns.

## 5. Conclusions and Future Perspectives

This review synthesizes the current understanding of how microplastics (MPs) alter soil physicochemical properties, microbial communities, and GHG dynamics. Across the studies examined, a coherent picture emerges: MPs are not inert bystanders in soil ecosystems but active modifiers of biogeochemical cycles. At the physicochemical level, fiber and film MPs modify soil pore architecture, bulk density, and water retention, with downstream consequences for aeration and microbial habitats. These structural changes interact with microbial responses: notably as shifts in taxon-level composition that increase Proteobacteria while reducing overall bacterial abundance and promoting fungal populations. The plastisphere, as a distinct microbial habitat on plastic surfaces, differs markedly from bulk soil and adds complexity to community dynamics. MP effects on carbon and nitrogen cycling are mediated through multiple pathways, including altered DOC, MBC, and EOOC, as well as modified nitrogen mineralization–immobilization dynamics. Speculative mechanisms such as microbial electron shuttling have been proposed to explain some of these changes, yet direct evidence for MP-mediated electron transfer remains limited and requires validation through in situ approaches. Similarly, effects on GHG emissions are highly context-dependent, varying with polymer type, concentration, particle size, aging status, soil properties, and experimental conditions. The same MP treatment can enhance, suppress, or exert negligible effects on emissions, and pooled estimates from meta-analyses—while indicating overall increases—should not be generalized to individual systems given that opposite or null responses are frequently reported under specific conditions.

These findings highlight critical research gaps. Most current evidence derives from laboratory or pot studies using MP concentrations 1–3 orders of magnitude above field-detected levels; future work must shift toward environmentally relevant doses, polymer mixtures, and weathering states under natural fluctuations. Long-term field studies tracking MP aging, fragmentation, and multi-year GHGs flux measurements are urgently needed to capture processes that short-term incubations cannot resolve. Equally critical is the move beyond single-factor exposures: real agricultural soils contain MPs alongside heavy metals, antibiotics, and pesticides, and factorial experiments are required to determine whether co-occurrence amplifies or mitigates individual MP effects. Emerging MP-derived low-molecular-weight degradation products, which may gradually integrate into labile SOC fractions, should be incorporated into future carbon models to avoid underestimating long-term soil responses. Addressing these gaps will refine understanding of MP–soil–climate interactions and inform evidence-based risk assessment and mitigation strategies.

## Figures and Tables

**Table 1:** The impact of MPs on soil properties.

Type	Size	Concentration	Soil Type	Duration	Soil Properties	Reference
PE	100 μm	28%	Clay	150 days	↓ TN, ON, TOC, DOC and pH; ↓ enzyme activities (catalase, manganese peroxidase, phenol oxidase, laccase, urease, and β-glucosidase); ↓ three aggregate-size fractions.	[47]
PE	<13 μm, <150 μm	5%	Clay	30 days	NS DOC; larger MPs ↑ bacterial community richness, diversity and abundance-based coverage on day 3 and ↓ on 30-day; smaller MPs ↑ bacterial and fungal community richness and diversity.	[48]
LDPE	<187.5 μm	0.01%, 0.1%, 1.0%	Silt Loam	30 days	NS DOC or MBC; low doses (0.01% and 0.1%) NS SOC decomposition; ↑ the *sga* and *abfA* genes but NS *manB*, *xylA*, *cex*, *lig* and *mnp* functional genes.	[49]
PET	5, 50, 100, 300–500, 800 μm and 2 mm	0.5%	——	90 days	↑ OC; changed microbial diversity, especially ↑ the microorganisms capable of degrading complex organics such as *Saprospiraceae*.	[50]
PE	6020 μm	0.1%, 0.3%, 0.9%	——	49 days	↓ WSA (>0.25 mm); ↓ WHC; NS WC; ↓ ammonium (8.1–12.9%); ↓ nitrifier abundance and genes encoding ammonium and hydroxylamine oxidation (*amoB*, *amoA*, *amoC*, and *hao*).	[51]
PE	<187.5 μm	1.0%	Paddy	42 days	NS DOC; NS NH_4_^+^-N and NO_3_^−^-N; ↓ *mcrA* gene and ↑ *pmoA* gene copies; NS functional genes encoding starch, hemicellulose, and lignin degradation.	[52]
PE, PLA	106 μm	0.01%, 0.1%	——	300 days	↓ pH; NS AP content; NS NH_4_^+^-N and ON contents; NS *nifH*, AOB*-amoA*, AOA*-amoA*, and *nirK*; NS C- and N-cycling functional bacterial groups.	[53]
PE	<0.5 mm	≈0.001%	——	——	MP-C to the SOC pool was approximately 1.59‰ (ranging from 0.05 to 14.24‰) indicating MP-C should not be neglected when estimating the soil C sequestration in the mulched agricultural fields.	[54]
PVC, PP, PE, PS, PET	100 μm	0.25%, 2%, 7%	Clay	15 days	↑ pH; ↑ SOC; NS TN; PE ↑ NH_4_^+^-N content but PP ↓ NH_4_^+^-N content; PP, PE, PS, and PET (7%) ↓ NO_3_^−^-N content; ↑ total microbial PLFAs.	[55]
PE	<187.5 μm	0.01%, 0.10%, 0.50%, 1.00%	Paddy	3, 15 and 40 years	↓ NO_3_^−^-N content; MP addition significantly affected the relative abundances of the *gdhA*, *amoA* and *nirS1* genes; NS major nitrifying or denitrifying bacterial genera.	[56]
PE	ball, powder, fiber and fragment	0.01%, 0.1%, 1%	Irrigated desert	2 years	The fragment- and fiber-shape MPs displayed more pronounced interfacial continuity with soil particles and induced greater soil bacterial α-diversity, relative to the powder- and ball-shape ones.	[35]
PES	<2 mm	0.1%, 0.3%, 1.0%	Clayey Nitisol	144 days	↓ SOM; ↑ cellulase and laccase activities; NS microbial communities while the bacterial community attached on PES fiber was more diverse than surrounding soils.	[57]
PES, PA, PP, LDPE, PET, PU, PS and PC	fibers, films, foams, and fragments	0.1%, 0.2%, 0.3%, 0.4%	Sandy Loam	28 days	Fiber-shape MPs hold soil water for longer; film-shape MPs ↓ soil bulk density; foam-shape MPs and fragment-shape MPs ↑ soil aeration and macroporosity; all shapes MPs ↓ soil aggregation by ~25%.	[32]
PLA, HDPE, Synthetic fibers	102.6 μm, 65.6 μm	0.1%	Sandy Loam	30 days	↓ pH (especially HDPE); NS SOM; ↓ MWD; the size distribution of water-stable soil aggregates was altered, suggesting potential alterations of soil stability.	[58]
PE, PVC, PHA	<5 mm	0.5%	——	60 days	PE and PVC ↓ OM degradation but PHA ↑ OM degradation; PE, PVC and PHA ↓ NO_3_^−^-N content (especially PE).	[59]
PA, PES, PE, PP, PET, PS	beads, fibers, fragments	0.2%, 2%	Sandy Loam	60 days	↓ BD (PE, PES, PET, PP and PS); ↓ WSA (PA and PES); ↑ evapotranspiration (~35% by PA and ~50% by PES); ↑ the general microbial metabolic activity (PA, PE and PES).	[40]
LDPE, PLA	4–10 mm, 50 μm–1 mm	1%	Sandy Loam	120 days	↑ pH; ↓ EC; PLA ↑ *Bacillus*, *Variovorax*, *Comamonadaceae* relative abundances; LDPE ↑Saccharibacteria relative abundance; medium size of LDPE and PLA ↑ the C:N ratio.	[38]
PP, PS	<250 μm	1%, 5%, 10%	Alluvial	56 days	↓ erosion resistance of coarse aggregates (>2 mm), and this negative effect became more pronounced as the concentration increased; ↓ stability of the water-stable soil aggregates.	[60]
LDPE, PBAT	50–200 μm, 200–500 μm, 0.63–1.2 mm	0.1%, 1.0%	Sandy Loam, Loamy	28 days	LDPE NS affects MBC neither in sandy loam soil nor in loamy soil; PBAT ↑ MBC both in sandy loam and loamy soil when added in large concentration (1%).	[61]
PP, PS	1 mm, 0.5 mm	1%	——	90 days	↓ pH; ↓ TP; ↓ NH_4_^+^-N; ↓ TN; ↓ SOM; ↑ Proteobacteria abundance on MPs surfaces in the top soil layer over the 90 days while an opposite trend was showed for the middle soil layer.	[62]
PP, PLA	~50 μm	0.5%, 1.5%	Paddy	41 days	↑ pH; ↑ FDAse activity; NS on soil bacterial diversity but altered soil bacterial richness; NS TN; ↑ TOC and DOC; PLA ↑ NH_4_^+^-N; PLA and PP (1.5%) ↓ NO_3_^−^-N (57.06 and 69.4%).	[63]
PE, PBAT, PLA	96–315 μm	0.5%	Loamy Clay	100 days	↑ pH; ↓ NH_4_^+^-N; ↓ NO_3_^−^-N; NS TN; NS bacterial community structure; PE and PLA NS on DOC; PE and PLA NS β-glucosidase activity but ↑ bacterial richness; PBAT ↑ DOC content; PBAT ↑ microbial biomass and ↓ β-glucosidase activity.	[64]
PE, PLA	25 μm	0.1%, 1%	Red soil; fluvo-aquic soil	120 days	PLA ↓ NH_4_^+^-N and NO_3_^−^-N; ↑ DOC and pH; MPs ↑ urease, sucrase, catalase and phosphatase activities; altered microbial community assembly.	[12]
PP, PLA	<5 mm	0.2%	Agricultural topsoil	2 years	NS total SOC; PLA ↓ lignin phenols (32%) and ↑ microbial-necromass contribution to SOC (35%); promoted fungal-necromass-associated C stabilization.	[65]
PE (virgin/UV-aged), PLA	<75 μm	0.5%, 1.5%	Agricultural topsoil	450 days	↓ MWD and GMD; shifted aggregates toward smaller fractions; ↑ DOM transformation, especially under PLA; microbial communities mediated DOM redistribution among aggregates.	[27]
LDPE, PBAT	<50–4000 μm	0.1–5.0%	Loamy sand	——	↑ WSA and water retention; ↓ hydraulic conductivity; 5% MPs ↑ splash erosion; NS BD, surface roughness and water repellency.	[46]
PE, PLA	10 μm	0.1%, 0.5%	Mollisol	180 days	PLA ↓ EOC (21.42–39.68%) and 0.5% PLA ↓ bulk SOC (6.79%); PE ↑ aggregate-associated SOC; PLA ↑ urease (25.21%) and β-glucosidase (47.46%).	[66]
PE, PLA	<75 μm	1%	Purplish agricultural soil	150 days	↓ available P (~15%); NS TP; ↓ phosphatase activities and *phoA*, *phoD* and *phoX* abundance; reduced P-cycling microbial interactions.	[67]
PS, PVC, PLA	150 μm	5%	Forest topsoil	60 days	PS ↓ TC/SOC (~3%); PVC and PLA ↑ TC/SOC (5–7%); all ↓ TN (3–10%) and ↑ NH_4_^+^-N (9–259%); altered soil enzyme activities.	[68]
PET, PVC	≥1 mm	1%	Farmland soil	80 days	Altered rhizosphere and plastisphere bacterial communities; PET plastisphere showed greater microbial diversity and network complexity; MPs enriched degradation- and metal-resistance-related functions.	[69]
PBAT, SBB, PE	~430 μm	0.11%	Sandy loam	8 weeks	NS soil nutrient availability, pH, DOC and microbial biomass; WHC differed among polymers, with PE highest and PBAT lowest.	[70]

Note: MPs—microplastics, PE—polyethylene, LDPE—low-density polyethylene, PS—polystyrene, PP—polypropylene, PA—polyamide, PVC—polyvinyl chloride, PLA—polylactic acid, PHA—polyhydroxyalkanoate, PET—polyethylene terephthalate, PES—polyethersulfone, PBAT—polybutylene terephthalate, SOM—soil organic matter, TOC—total organic C, SOC—soil organic carbon, MBC—microbial biomass carbon, DOC—dissolved organic carbon, TN—total nitrogen, TP—total phosphorus, AP—available P, ON—organic N, EC—electrical conductivity, BD—bulk density, WSA—water-stable aggregates, WC—water conductivity, WHC—water-holding capacity, MWD—mean weight diameter, SBB—starch-based biodegradable plastic, EOC—easily oxidizable organic carbon, DOM—dissolved organic matter, GMD—geometric mean diameter, TC—total carbon. ↑ increase, ↓ decrease, NS—no statistically significant change, —— information not reported in the original study. Experimental concentrations reported in this table refer to the nominal exposure levels used in the cited studies and should not be interpreted as representative concentrations in field soils.

**Table 2:** The impact of MPs on GHG emissions.

Type	Size	Concentration	Soil Type	Duration	GHG Emissions	Reference
PE	<13 μm, <150 μm	5%	Clay	30 days	↓ N_2_O emissions on day 3 but ↑ N_2_O emissions on 30-day.	[48]
LDPE	<187.5 μm	0.01%, 0.1 and 1.0%	Silt Loam	30 days	High dose (1.0%) ↑ the production of CO_2_ (15–17%).	[49]
PET	5, 50, 100, 300–500, 800 μm and 2 mm	0.5%	——	90 days	5 μm MP ↑ CO_2_ and N_2_O emissions after 7 day; 300–800 μm ↑ CO_2_ emissions after 3 day.	[50]
PE	6020 μm	0.1%, 0.3% and 0.9%	——	49 days	↓ N_2_O emission (16.3–34.3%).	[51]
PE	<187.5 μm	1.00%	Paddy	42 days	↓ CH_4_ emissions (16.1–16.9%) in acidic or alkaline soil but NS in neutral soils; NS CO_2_ emissions.	[52]
PE, PHBV	PE: 40–48 μm; PHBV: 1–15 μm	100 kg ha^−1^ (~0.01%)	Eutric Cambisol, sandy clay loam	One growing season	PE ↓ cumulative N_2_O emissions by ~67%; PHBV NS.	[106]
PVC, PP, PE, PS, PET	100 μm	0.25%, 2%, 7%	Clay	15 days	↑ N_2_O, CO_2_ and CH_4_ emissions; PE may cause most GHG emissions.	[55]
PE	<187.5 μm	0.01%, 0.10%, 0.50%, 1.00%	Paddy	3, 15 and 40 years	Low doses MPs (0.01% and 0.1%) NS N_2_O emissions but high doses (0.5% and 1.0%) ↑ N_2_O.	[56]
PE, PVC, PHA	<5 mm	0.5%	——	60 days	PE and PHA ↑ CH_4_ and NH_3_ emissions by 7.9–9.1% and 20.9–33.9%, respectively; PVC ↓ CH_4_ and NH_3_ emissions by 6.6% and 30.4%, respectively; PE and PVC ↑ N_2_O emissions while PHA ↓ N_2_O emissions by 11.8%.	[59]
LDPE, PBAT	50–200 μm, 200–500 μm, 0.63–1.2 mm	0.1%, 1.0%	Sandy Loam and Loamy	28 days	LDPE NS CO_2_ emissions but PBAT with high concentration ↑ CO_2_ emissions (13–57%); Small PBAT particles ↑ CO_2_ emissions (10–13%) compared to large ones.	[61]
HDPE	250–1000 μm	0.23%, 0.46%	Loam	730 days	Higher dose generally ↓ soil CO_2_ flux after ~166 days; lower dose showed no consistent trend.	[43]
PLA	50 μm	1%, 10%	Forest and farmland Acrisols	168 h	↑ CO_2_, greatest at 10% PLA under warming; N_2_O showed a nonlinear response, peaking at 1% PLA and declining at 10%.	[107]
PLA	<2–500 μm	0.1%, 1%, 5%	Eight agricultural soils	70 days	Soil-derived CO_2_ priming was soil- and time-dependent; alkaline soils shifted from early positive to later negative priming, whereas acidic soil remained positive.	[108]
PE, PET, PBS, PLA	150 μm	0.5%	Flooded paddy soil	60 days	PLA ↑ cumulative CH_4_ (264%) and CO_2_ (27.3%); PE and PET ↓ CH_4_ and CO_2_; PE ↑ cumulative N_2_O (93.1%); PLA showed NS effect on N_2_O.	[103]
PS, PVC, PLA	150 μm	5%	Forest topsoil	60 days	All MPs ↑ cumulative CO_2_ (16–129%); PVC ↑ CH_4_ (39%), whereas PS and PLA ↓ CH_4_ (65% and 36%); PS and PLA ↑ N_2_O (315% and 421%), whereas PVC ↓ N_2_O (30%).	[68]
LDPE, PBS, PLA, PLA/PHA	~100 μm	2%	Decatur silt loam	193 days	Polymer-specific SOC priming: LDPE induced negative priming, whereas PLA, PBS and PLA/PHA induced positive priming; PLA/PHA showed the greatest plastic-C mineralization.	[74]
PE, PVC, PLA, PBAT	150–300 μm	0.5%	Heavy-metal-contaminated farmland soil	90 days	All MPs ↓ cumulative CH_4_ and N_2_O; PLA/PBAT showed stronger reductions; PBAT ↑ CO_2_ under one planted-soil condition.	[109]
PE, PLA	150–180 μm	~0.1%, ~0.3%, ~0.5%	Calcic Cambisol, silty loam	2 years	↑ soil C loss via respiration; respiratory ^13^C loss ↑ 7.9% under PE and 14.3% under PLA; soil ^13^C retention decreased.	[110]

Note: MPs—microplastics, PE—polyethylene, LDPE—low-density polyethylene, PS—polystyrene, PP—polypropylene, PVC—polyvinyl chloride, PLA—polylactic acid, PHA—polyhydroxyalkanoate, PET—polyethylene terephthalate, PBAT—polybutylene terephthalate, HDPE—high-density polyethylene, PBS—polybutylene succinate, PLA/PHA—polylactic acid/polyhydroxyalkanoate blend. GHG—greenhouse gas, ↑ increase, ↓ decrease, NS—no statistically significant change, —— information not reported in the original study. Experimental concentrations reported in this table refer to the nominal exposure levels used in the cited studies and should not be interpreted as representative concentrations in field soils.

## Data Availability

The data that support the findings of this study are available from the corresponding author upon reasonable request.
